# The Role of a Sustainable Planetary Health Diet in the Prevention of Non-Communicable Diseases and Cause-Specific Mortality: A Narrative Review

**DOI:** 10.3390/foods14223909

**Published:** 2025-11-15

**Authors:** Dorota Różańska, Bożena Regulska-Ilow

**Affiliations:** Department of Dietetics and Bromatology, Wroclaw Medical University, 50-556 Wroclaw, Poland; bozena.regulska-ilow@umw.edu.pl

**Keywords:** Planetary Health Diet, plant-based food, mortality, cardiovascular disease, cancer, non-communicable diseases

## Abstract

Taking into account both the health and environmental aspects of food, the EAT-Lancet Commission proposed a healthy reference diet (Planetary Health Diet—PHD). The aim of this narrative review is to summarize the results obtained in epidemiological studies on the association between the PHD and risk factors, non-communicable diseases, and cause-specific mortality. The literature search was conducted in February 2025 and was based on the PubMed electronic database. The results of this review are divided into four parts, which include the results of cohort studies, cross-sectional studies, case–control studies, and meta-analyses. This review, showing what types of studies have been conducted so far, allows for a summary of the current knowledge of the relationship between the PHD and risk factors, non-communicable diseases, and cause-specific mortality. Cohort studies provided most of the results, which confirmed that higher adherence to the PHD has a beneficial effect on human health, especially taking into account the lower risk of diabetes, cardiovascular disease (CVD) and CVD mortality, cancer and cancer mortality, as well as all-cause mortality. However, it is concluded that the association between the PHD and stroke, different types of stroke, hypertension, dyslipidemia, and some specific types of cancer must be confirmed. Specifically, randomized controlled trials should be conducted, as, to our knowledge, there is a lack of these types of studies to date. Such studies should be conducted in different regions using the Planetary Health Diet adapted to the local, cultural, geographical, and demographical aspects of a particular region.

## 1. Introduction

Non-communicable diseases, mainly cardiovascular diseases (CVDs) and cancer, remain the main cause of death worldwide [1]. It is well known that a healthy diet high in plant food and with a low intake of red meat has a positive impact on human health. One of the most important and earliest studies (baseline conducted between 1958 and 1964) that proved this association is “The Seven Countries Study”. The results observed after 25 years of follow-up showed that the coronary heart disease death rate was negatively correlated with vegetable food intake, while there was a positive correlation with animal food intake (excluding fish) [2]. The dietary pattern which was observed in the 1950s and 1960s in Greece and Italy is now known as the “Mediterranean Diet” (MD). It is characterized by low content of saturated fatty acids, whereas the main source of fats is olive oil. In the traditional MD, the intake of plant food (fruit, vegetables, legumes, and cereal) was high, whereas the intake of dairy and meat products was low. There was also a moderate consumption of fish and wine [3]. Many other studies on the MD and different health conditions have been conducted since “The Seven Countries Study”. Its benefits have not only been proven for CVD [4,5,6,7], but also, for example, for cancer [8,9,10] or age-related cognitive disorders [11,12]. The European Society of Cardiology recommends the Mediterranean Diet for cardiovascular disease prevention; however, it is also emphasized that a shift from a more animal-based to a plant-based food pattern may have a favorable impact on reducing the risk of CVD [13]. A healthy eating pattern is also an important tool in cancer prevention; based on The American Cancer Society guidelines, it is recommended to follow a diet rich in plant food (colorful vegetables and fruits, and also legumes and whole grains), whereas the intake of red and processed meat, sugar-sweetened beverages, highly processed foods, and refined grains products should be limited or excluded [14]. Notably, in both guidelines, it was also highlighted that a more plant-based food dietary pattern with a limited amount of animal-based food may also be associated with improved environmental sustainability [13,14].

Food production is one of the major drivers of global environmental change. According to the EAT-Lancet Commission report published in 2019, more than 820 million people lack sufficient food globally. In addition, an even larger number of the population consumes a low-quality diet or too much food, which leads to diet-related disorders such as obesity and non-communicable diseases. It is predicted that, by 2050, the problem with non-communicable diseases will increase. At the same time, as the effect of food production, the stability of the Earth system will be reduced. Depending on the type of food, its production has a different impact on environmental aspects, such as greenhouse gas (GHG) emissions, nitrogen and phosphorus pollution, biodiversity loss, and water and land use [15]. Animal food production affects the environment much stronger than plant food. The greatest GHG emissions (per serving) is caused by ruminant meats (beef and lamb), but the GHG emissions caused by pork or poultry are lower. Notably, seafood, depending on how it is caught, may cause higher GHG emissions than pork or poultry [15,16]. This happens for trawling fishery and recirculating aquaculture [16]. GHG emissions caused by dairy and egg production is lower than those caused by meat or fish, but higher than those caused by cereals, fruits, vegetables, nuts, roots, or legumes [15,16]. The greatest impact on land use is also caused by the production of ruminant meat (per serving), and it is much higher than in case of pork, chicken, dairy, or eggs. Moreover, the production of all types of meat, dairy, and eggs has a high impact on acidification potential and eutrophication potential (ruminant meat is the highest, chicken and eggs is the lowest) [15].

Taking into account both the health and environmental aspects of food, the EAT-Lancet Commission proposed a healthy reference diet (Planetary Health Diet). It is a plant-based diet that allows for the consumption of small amounts of products derived from animals. The possible ranges for each food group are given for an intake of 2500 kcal/day [15,17]. A planetary health plate is divided into two halves, the first of which are vegetables and fruits, meaning that they should consist by volume approximately half a plate. The second half of the plate is sub-divided, taking into account the contribution of calories. The largest part is made up of whole grains, plant sources of protein (nuts and legumes), and unsaturated plant oils. Moderate amounts of animal protein sources are also included, but they are optional. In this diet, the content of refined grains, as well as highly processed foods and added sugars, is limited. Unsaturated fatty acids are preferred instead of saturated fatty acids [17]. It was estimated that changes in dietary patterns from those currently observed toward healthy diets could prevent about one fifth (19–23.6%) of total deaths amongst adults globally, which means about 11 million deaths per year. However, it is a significant challenge, as the global intake of unhealthy foods should be reduced by more than 50%, while the intake of healthy foods should be increased by more than 100%. It is important to note that substantial differences exist across regions, for example, the intake of red meat in North America was about 640% of the reference intake; in Europe, central Asia, Latin America, and Caribbean, it was about 450% of the reference intake; in the East Asia Pacific, it was about 350%; while in the Middle East and North Africa, it was about 120%. The intake of red meat in the Sub-Saharan African region was consistent with the reference intake, but in South Asia, it was about 50%. Conversely, the consumption of plant foods, such as nuts, legumes, fruits, and whole grains, was below the recommended level globally and in each region (with different proportions). In the case of vegetables, only in the Middle East and North Africa were the intake close to the recommended level. In other regions, the intake was lower [15,17].

The concept of the PHD was proposed in 2019. This narrative review was prepared to show what type of studies have been conducted so far, as well as the PHD’s association with risk factors, main non-communicable diseases, and cause-specific mortality. A wide variety of studies have been included to provide an overall summary of the issue, which may reveal some directions for future research, due to a lack of data or insufficient data in some respects. The aim of this narrative review was to summarize the results obtained in epidemiological studies on the association between the Planetary Health Diet and risk factors, non-communicable diseases, and cause-specific mortality.

## 2. Materials and Methods

This paper is a narrative review intending to discuss the association between the Planetary Health Diet and non-communicable diseases, as well as its risk factors and cause-specific mortality. A literature search was conducted in February 2025 and was based on the PubMed electronic database. It was carried out in three steps (Supplementary Materials, Figure S1). At the beginning, the literature search was based on the following words: “EAT-Lancet” or “Planetary Health Diet”, appearing in title/abstract together with one of the following words: “cancer” or “cardiovascular disease” or “hypertension” or “diabetes” or “mortality” or “obesity” or “risk factor” or “risk factors”. Then, review papers and repetitive papers were excluded. Only full-text and English language papers were chosen. Finally, abstracts and full-texts were read to be sure that the remaining articles aligned with the scope of this paper. Taking into account that different types of studies have various strengths of evidence, this review was divided into four parts, where the results of cohort studies, cross-sectional studies, case–control studies, and meta-analyses are summarized. No original studies were excluded because this narrative review aims to summarize the current knowledge on the relationship between the PHD and risk factors, non-communicable diseases, and cause-specific mortality.

## 3. Results

### 3.1. Cohort Studies

The results of cohort studies published in thirty-four publications are presented in Table 1 [18,19,20,21,22,23,24,25,26,27,28,29,30,31,32,33,34,35,36,37,38,39,40,41,42,43,44,45,46,47,48,49,50,51]. A vast majority of these studies showed beneficial health effects associated with higher adherence to the Planetary Health Diet (PHD). Higher adherence to the PHD was associated with higher HDL-cholesterol (high-density lipoprotein cholesterol) level [18] and lower risk of hypertension [19]. In four studies conducted in Sweden, the United Kingdom, Denmark, and China, the Planetary Health Diet was associated with a lower risk of diabetes [20,21,22,23]. There were also two analyses carried out using the data from UK Biobank, where the Planetary Health Diet was found to be inversely correlated to the risk of metabolic-dysfunction-associated steatotic liver disease [24] and both chronic and severe liver disease [25]. The results regarding anthropometric parameters are inconsistent. In a Danish study conducted by Langmann et al. [26], the PHD was associated with lower waist circumference at follow-up, with a lower risk of obesity and elevated waist circumference, whereas it was not associated with body weight at follow-up. Suikki et al. [27] did not observe significant associations between the PHD and changes in body weight, body mass index, and waist circumference during the follow-up among participants from Finland. Moreover, Finnish authors found that the genetic susceptibility to obesity and anthropometric parameters was not influenced by the PHD [28]. Masip et al. [29] also showed that the PHD did not mediate or moderate obesity polygenic risk; however, it was associated with a lower body mass index, waist circumference, and body fat percentage.

A majority of cohort studies confirmed that there was a favorable relationship between the PHD and reduced risk of cardiovascular disease [23,30,31,32], coronary events [33], coronary heart disease [30,32], myocardial infarction [31], heart failure [34], and stroke [30,31]. However, in some studies, this association was not observed. For example, in an analysis based on a Danish Diet, Cancer, and Health cohort with 55,016 adults, the Planetary Health Diet was only associated with a reduced risk of subarachnoid stroke, while no significant relationship was found for total stroke, ischemic stroke, or intracerebral hemorrhage [35]. In four other studies conducted in Spain, the UK, Switzerland, and France, an association between the PHD and cardiovascular events was not observed [36,37,38,39]. In contrast, in many studies, higher adherence to the PHD was inversely associated with the risk of CVD mortality [40,41,42,43,44].

The diet proposed by the EAT-Lancet Commission was also analyzed, taking into account its association with cancer. It was observed that this dietary pattern was related to a lower risk of cancer overall [37,44,45], including, but not exclusively limited to, lung cancer [46,47], colorectal cancer [48], head and neck cancers, oral cavity, and pharyngeal cancers [49]. There was also a lower risk of cancer among women in a study conducted by Berthy et al. [39]; however, such an association was not observed in the whole group of participants. Moreover, a majority of cohort studies found that there was a lower risk of cancer mortality among participants with higher adherence to the PHD [40,41,42]. Such an association was observed also for lung cancer mortality [46,47]. However, Pitt et al. [43] in a study conducted in Sweden with 22 years of follow-up did not observe the association between the PHD and cancer mortality. In a study conducted in Italy this dietary pattern was not associated with breast cancer risk; conversely, it has been observed that the higher values of the EAT-Lancet Index were related to the higher levels of C-reactive protein. Nevertheless, the authors admitted that not all of the EAT-Lancet Index components were taken into account in calculations (soy foods and nuts), as these were not included in the food frequency questionnaire which was used in the study [50]. In another study conducted in a sample from the German population, the PHD was not significantly associated with inflammatory biomarkers, such as high-sensitivity C-reactive protein and chemerin [51].

All of the studies where the all-cause mortality was assessed in relation to the PHD showed an inverse relationship between them [23,37,38,40,41,42,43,44,45].

### 3.2. Cross-Sectional Studies

Table 2 shows the results from cross-sectional studies. In all of them, some associations between the Planetary Health Diet and selected CVD risk factors were found [52,53,54,55,56,57,58,59]. In two Brazilian studies, the PHD was associated with lower levels of total and LDL-cholesterol (low-density lipoprotein cholesterol), while no association was found with HDL-cholesterol and triglycerides [52,53]. However, Frank et al. [54] conducted a study on cardiometabolic risk factors with cut-off points, such as the metabolic syndrome criteria from 2009, and observed that the PHD was associated with higher levels of HDL-cholesterol, lower levels of fasting triglycerides, and a lower predicted probability of having low HDL-cholesterol level. This dietary pattern was associated with lower systolic blood pressure in the study conducted by de Oliveira Neta et al. [52] and with lower systolic and diastolic blood pressure in the study conducted by Cacau et al. [53]. Conversely, Frank et al. [54] did not observe the association between the PHD and systolic or diastolic blood pressure; however, the predicted probability of having high blood pressure decreased with higher adherence to this dietary pattern. Moreover, in another study conducted in Australia, participants with high blood pressure (ever and current) had lower adherence to the EAT-Lancet reference diet than those without this abnormality [55]. This dietary pattern was not associated with fating blood glucose [52,54] and with HOMA-IR [53]; however, de Oliveira Neta et al. [52] observed a lower risk of self-reported type 2 diabetes among those who followed the PHD. McDowell et al. [55] observed lower adherence to the PHD among participants with diabetes (ever) in comparison to those without diabetes. There is also some data about the association between the Planetary Health Diet and anthropometric parameters. Higher adherence to the PHD was also associated with a lower waist circumference [54,56] and body mass index [56,57]. Moreover, the results presented by Cacau et al. [56] show that this dietary pattern was related to a lower risk of overweight and obesity and increased or substantially increased waist circumference. Ambroży et al. [58] also observed an association between the Planetary Health Diet and excessive body mass. In another study conducted among Iranian adults, the PHD was associated with a lower prevalence of metabolic syndrome and abdominal obesity and with a lower prevalence of low HDL-cholesterol levels [59].

### 3.3. Case–Control Studies

During the literature search described above, only one case–control study was found. Mohammadi et al. [60] conducted a study where the relationship between adherence to the Planetary Health Diet and colorectal cancer was assessed. It was observed that the risk of colorectal cancer in the third tertile of the Planetary Health Diet Index was almost 60% lower than in the first tertile (*p* = 0.028).

### 3.4. Meta-Analysis

Only one meta-analysis was found based on the inclusion criteria described above. It was conducted based on the data of over 2.21 million participants from 28 publications. Its results support the evidence about the beneficial health effects of the PHD, showing lower risks of diabetes, CVD (combined with mortality), cancer (combined with mortality), and all-cause mortality [61].

## 4. Discussion

In this review, we collect the results of studies where the relationship between the Planetary Health Diet and non-communicable diseases, its risk factors, and cause-specific mortality was analyzed. Most of these results came from cohort studies conducted in different countries with many years of follow-up. Most of them confirmed that higher adherence to the diet, which was proposed by the EAT-Lancet Commission, has a beneficial effect on human health, especially taking into account the lower risks for diabetes [20,21,22,23], cardiovascular diseases [23,30,31,32,33,34], cancer [37,39,46,47,48,49], and overall and/or cause-specific mortality [23,37,38,40,41,42,43,44,45,46,47]. Although some researchers did not find statistically significant associations, the results of the meta-analysis published by Liu et al. [61] confirmed the advantages of following this dietary pattern. Most of the results from cross-sectional studies showed that the PHD was associated with lower values of waist circumference [54,56] and body mass index [56,57], as well as with lower risk of obesity and abdominal obesity [56,58,59]. Only one case–control study was found, and its results showed a lower risk of colorectal cancer in the group with the highest adherence to the Planetary Health Diet [60].

The association between the PHD and health is a result of its composition. Bui et al. [44] found that, among 15 elements included in the PHD index, higher intakes of tubers, red/processed meats, eggs, added saturated and trans fats, added sugars, and sugar from fruit juices were associated with a higher risk of total mortality, whereas higher intakes of whole grains, whole fruits, poultry, nuts, soy foods, and added unsaturated fats were associated with a lower risk of total mortality. It is well-known that plant foods are abundant in dietary fiber, antioxidants, and anti-inflammatory compounds. A higher intake of these compounds is associated with lower prevalence of non-communicable diseases [62,63,64,65,66,67]. Higher adherence to the PHD is also positively associated with potassium intake [57], which may partially explain its role in hypertension [68] or stroke [68,69]. The recommended foods, such as vegetables, fruits, whole grains, and legumes, generally have low glycemic index values. The association between a high glycemic index and a higher prevalence of diabetes [70,71], metabolic syndrome [71,72], CVD [71,73], or some types of cancer [74,75,76] was found in different studies. On the other hand, the intake of saturated fatty acids and red meat is discouraged. It is recommended to decrease the intake of saturated fatty acids below 10% of total daily energy intake in order to lower the risk of CVD [13]. Both for CVD and cancer prevention, the intake of red meat should be limited to up to a maximum of 350–500 g per week, and the intake of processed meat should be even more limited or completely eliminated [13,77]. Red meat is a source of saturated fatty acids, but it was also classified by the International Agency for Research on Cancer (IARC) as a group 2A carcinogen, while processed meat was classified as a group 1 carcinogen [78]. The reference Planetary Health Diet only allows for a maximum 28 g/day of beef, lamb, and pork (when diet energy is 2500 kcal/day) [17]. Avoiding the consumption of processed food may also be associated with lower sodium intake. This may be another explanation of lower risk of hypertension [79], stroke [80], or CVD [81]. High sodium intake is also associated with gastric cancer [82]. Nonetheless, in this review, we did not find studies on the association between the PHD and gastric cancer.

A shift to the PHD may have a positive effect on human health, but not only because of its composition; another explanation is that higher dietary GHG emissions are associated with a higher risk of cancer or CVD and mortality [83,84], so a diet that causes lower GHG emissions will also contribute to lower exposure to them in the future. The results of a French cohort study showed that diets with low overall environmental pressures are associated with a lower risk of cancer, coronary heart disease, and diabetes [85]. Observations from the study above are in accordance with the previous results from The European Prospective Investigation into Cancer and Nutrition (EPIC) study, where it was found that higher greenhouse gas emissions (fourth quartile vs. first quartile) increased the risk of all-cause mortality and mortality caused by coronary heart disease, cardiovascular disease, and cancer. The same associations were observed for higher land use contributions. Moreover, higher greenhouse gas emissions increased the risk of cancer and specific types of cancer such as bladder, renal, pelvis, ureter and other urinary organs, breast, esophagus, kidney, larynx, lung, skin melanoma, stomach, and thyroid. For higher land use contributions, even more associations were found (such as colorectum, liver, pancreas, and prostate cancer) [86].

Higher GHG emissions are associated with climate change, and climate change is associated with extreme heat and poor air quality, which may be associated with cardiovascular health [87]. Based on the data from the Global Burden of Diseases Study (2019), an increasing trend of the global burden of high-temperature-related non-communicable diseases was observed; the main contributor was CVD [88]. Higher air pollution (increased levels of fine particulate matter—PM_2.5_, large particulate matter—PM_10_, ozone, and nitrogen dioxide), caused by different climate-mediated events, may also increase the risk of different cardiovascular outcomes (for example, hypertension, arrhythmia, stroke, ischemic heart disease, or CVD mortality). There are many processes that may be involved in this association, such as inflammation (for example, because of the release of inflammatory cytokines or oxidized lipids), the depletion of endogenous antioxidants, oxidative stress, alterations in vascular and autonomic tone after exposure to air pollution, or even transcriptional and epigenetic reprogramming as a result of prolonged exposure to inhalational PM_2.5_ [87]. Air pollution was also found to be associated with an increased risk of lung cancer in different populations [89,90,91]. It may be caused by different mechanisms involving inflammatory injury, reactive oxygen species production, and oxidative damage to DNA, leading to the genotoxic and mutagenic effects as a result of long-term exposure to air pollution [91].

It was estimated that changing current dietary patterns to those proposed by the EAT-Lancet Commission can lead to the reduction in the number of deaths. According to the data presented by Gu et al. [92], a shift to the ideal PHD could prevent more than 21 million premature deaths among adults per year (39% of all-cause deaths in 2019). Based on the data from the EPIC study, it was estimated that, thanks to the shift to the EAT-Lancet reference diet, the number of deaths could be reduced by even up to 63% in a 20-year risk period [86].

The composition of the Planetary Health Diet is beneficial both for human health and the environment. However, actions focused on its implementation must be appropriate for individual regions, as it is known that there are different deviations from the Planetary Health Diet in different regions of the world [15,17]. A study conducted based on the data from 147 countries showed that Gross Domestic Product per capita is associated with food group consumption; in some countries, financial assistance is required to improve diet quality regarding the PHD [93]. Therefore, different countries need different dietary modifications [94,95]; in some cases, especially in low-income countries, it may be a significant challenge [95]. The costs of the EAT-Lancet diet was found to be higher than the national average expenditure on food in Indonesia, but lower than the national average in urban areas in Indonesia [95]. The costs of the EAT-Lancet diet were also lower than the typical Australian diet in metropolitan areas [96]. The PHD is a conception of a diet based on food groups, but it is emphasized that it does not imply that the global population should eat exactly the same food, nor does it prescribe an exact diet. The food groups and ranges of food intakes were proposed through empirical calculations. Local interpretation and adaptation of this reference diet is necessary, taking into account the cultural, geographical, and demographical aspects of the population and individuals [17]. A German study conducted by Kersting et al. [97] is a good example that shows, in practice, how dietary changes can make a diet more similar to the PHD.

## 5. Limitations

During the literature review, some important limitations became apparent; these need to be taken into account during future studies and analyses. Firstly, different authors used different scoring systems to estimate adherence to the Planetary Health Diet. The most popular were the indices proposed by the following: Knuppel et al. [98], with scoring ranging from 0 to 14 points; Stubbendorff et al. [42], with scoring from 0 to 42 points; Cacau et al. [99], with scoring from 0 to 150 points; and Bui et al. [44], with scoring from 0 to 140 points. Secondly, some authors were not able to exactly calculate the originally given index due to a lack of data in dietary interviews, and, therefore, the possible maximum value slightly differed from the original. For example, Sotos-Prieto et al. [31] used the index proposed by Bui et al. [44], as there was a lack of data about the unsaturated fatty acid component; the maximum possible value was 130 points. Karavasiloglou et al. [37] and Quartiroli et al. [50] used the method similar to Knuppel et al. [98], but because of some missing data, the maximum possible values were 11 and 12 points, respectively. Martins et al. [38] used the method described by Stubbendorff et al. [42], but the maximum possible index value in this study was 39 points. Another aspect that should be noted is the lack of randomized controlled trials in this topic, according to our knowledge. The PHD was proposed in 2019; there was probably not enough time to conduct and/or publish the results of this type of study. Randomized controlled trials are the most scientifically rigorous method for hypothesis testing and have one of the highest levels of evidence, closely followed by meta-analyses [100,101]. This form of study would enrich our knowledge and provide new and proven evidence. Good examples of randomized trials on diet are The Dietary Approaches to Stop Hypertension trial [102] and The PREDIMED (PREvención con DIeta MEDiterránea) study [103]. On the other hand, it is important to note that most of the available data was from cohort studies conducted in different populations. Cohort studies are analytical, observational studies where temporal association is most clearly delineated; based on the results, it is possible to build evidence toward causality [100,104].

## 6. Conclusions

Based on the literature review, it can be seen that the Planetary Health Diet proposed by the EAT-Lancet Commission has a beneficial association with human health. There is strong evidence that the PHD is associated with a lower risk of diabetes, CVD and CVD mortality, cancer and cancer mortality, and all-cause mortality. However, the association between stroke (and its variants) and the PHD must be confirmed in other studies, as not all of the results are consistent. More studies with a high level of evidence are needed to confirm the association between the PHD and hypertension or dyslipidemia. Despite the association between the PHD and the risk of total cancer and cancer mortality observed by many authors, more studies are needed in relation to specific types of cancer, for example, breast, colorectal, prostate, gastric, and other diet-related cancers. Some studies presented in this review calculated the PHD index by excluding some foods due to a lack of them in food frequency questionnaires. Randomized controlled trials regarding this topic should be conducted; this would equate to a reduction in the number of confounding factors and would provide data with a high level of evidence. These studies should be conducted in different global regions using the Planetary Health Diet adapted to the local cultural, geographical, and demographical aspects of the specific region.

## Figures and Tables

**Table 1:** Main results from cohort studies concerning the association between the Planetary Health Diet with non-communicable diseases, risk factors, and cause-specific and total mortality.

References	Time of Follow-Up	Characteristics of the Study Group	Results: The Planetary Health Diet vs.:
Morcel et al. [18]	10–14 years	N = 215; age 21–32 years at follow-up, Belgium, France, Italy, Spain	HDL-cholesterol level (↑)
Lei et al. [19]	93,058 person-years	N = 11,402; age 40.8 ± 14.0 years *, China	risk of hypertension (↓)
Zhang et al. [20]	24.3 years	N = 24,494; age 58.1 ± 7.7 years *, Sweden	risk of diabetes (↓)
Xu et al. [21]	10.0 years	N = 59,849; age 40–69 years * (55.91 ± 8.14), UK Biobank	risk of diabetes (↓)
Langmann et al. [22]	15.0 years	N = 54,232; age 50–64 years *, Denmark	risk of diabetes (↓)
Cai et al. [23]	9.86 years	N = 16,029, age 43.8 ± 14.6 years *, China	risk of diabetes, mortality, CVD (↓)
Wu et al. [24]	11.6 years	N = 105,752; age 39–72 years *, UK Biobank	risk of metabolic dysfunction-associated steatotic liver disease (↓)
Li et al. [25]	13.65 years	N = 459,502; age 56.53 ± 8.09 years *, UK Biobank	risk of chronic and severe liver disease (↓)
Langmann et al. [26]	5 years	N = 44,194; age 50–64 years *, Denmark	follow-up WC, risk of obesity, risk of elevated WC (↓) follow-up body weight (–)
Suikki et al. [27]	7 years	N = 4371; age 30–74 years *, Finland	changes in body weight, BMI, WC (–) during the follow-up
Suikki et al. [28]	7 years	N = 2942; age 53 ± 13 years *, Finland	lack of mediating or moderating role in the associations between genetic susceptibility to obesity and anthropometric parameters
Masip et al. [29]	6 years	N = 7037, age 55.6 ± 7.7 years *, Canada	BMI, WC, body fat percentage (↓) lack of mediating or moderating role in the obesity polygenic risk
Sawicki et al. [30]	approx. 30 years	N = 62,919 women from NHS I (age 30–55 years), 88,535 women from NHS II (age 25–42 years), and 42,164 men from HPFS (age 40–75 years), USA	risk of CVD, CHD, total stroke (↓)
Sotos-Prieto et al. [31]	9.4 years	N = 118,469; age 40–69 years *, UK Biobank	risk of CVD, myocardial infarction and stroke (↓)
Colizzi et al. [32]	15.1 years	N = 35,496; age 20–70 years *, Netherlands	risk of CVD and CHD (↓)
Zhang et al. [33]	24.9 years	N = 23,877; age 57.9 ± 7.7 years *, Sweden	risk of coronary events (↓)
Zhang et al. [34]	25.0 years	N = 23,260; age 57.8 ± 7.6 years *, Sweden	risk of heart failure (↓)
Ibsen et al. [35]	15 years	N = 55,016; age 50–64 years *, Denmark	risk of subarachnoid stroke (↓) risk of stroke, ischemic stroke, intracerebral hemorrhage (–)
Guzmán-Castellanos et al. [36]	11.5 years	N = 18,656; age 38 ± 12.1 years *, Spain	risk of CVD (–)
Karavasiloglou et al. [37]	10.49 years (cancer) 11.98 years (major CV events) 11.98 years (mortality)	N = 473,836–448,053 (depending on the outcome); age 56.50 ± 8.08 years *, UK Biobank	risk of cancer and all-cause mortality (↓) major cardiovascular events (–)
Martins et al. [38]	7.9 ± 2.0 years	N = 3866; age 35–75 years *, Switzerland	all-cause mortality (↓); cardiovascular events (–)
Berthy et al. [39]	8.1 years	N = 62,382; age 51.0 ± 10.2 years *, France	risk of cancer and CVD combined among low consumers of alcohol (↓) risk of cancer among females (↓) risk of cancer and CVD combined, and separately (–)
Ye et al. [40]	23.4 years	N = 57,078; age 45–74 years *, Chinese cohort from Singapore	risk of all-cause mortality, CVD mortality, cancer mortality, respiratory disease mortality (↓)
Han et al. [41]	8.50 years	N = 30,521; age 47.02 ± 17.01 years *, USA	risk of all-cause mortality, CVD mortality, cancer mortality, other-cause mortality (↓)
Stubbendorff et al. [42]	20 years	N = 22,421; age 45–73 years *, Sweden	all-cause mortality, cancer mortality, cardiovascular mortality (↓)
Pitt et al. [43]	22 years	N = 68,175; adults and elderly, Sweden	risk of all-cause mortality, cardiovascular mortality, cancer mortality (–)
Bui et al. [44]	34 years	N = 66,692 women from NHS I (age 30–55 years), 92,438 women from NHS II (age 25–42 years), and 47,274 men from HPFS (age 40–75 years), USA	risk of all-cause mortality, CVD mortality, cancer mortality, respiratory diseases mortality, neurodegenerative diseases mortality (↓), and risk of infectious diseases among women (↓)
Mangone et al. [45]	15.6 years	N = 47,749; mean age 50.5 years *, Italy	risk for overall mortality and cancer incidence (↓)
Liu et al. [46]	9.47 years	N = 175,214; age 58.09 ± 8.03 years *, UK Biobank	risk of lung cancer incidence and mortality (↓)
Xiao et al. [47]	8.82 ± 1.95 years	N = 101,755; age 65.5 ± 5.7 years *, USA	risk of lung cancer incidence and mortality (↓)
Ren et al. [48]	8.82 years	N = 98,415; age 65.52 ± 5.73 years *, USA	risk of colorectal cancer (↓)
Ren et al. [49]	8.84 years	N = 101,755; age 65.5 ± 5.7 years *, USA	risk of head and neck cancers, oral cavity and pharyngeal cancers (↓)
Quartiroli et al. [50]	22.6 years	N = 9144; age 35–69 years *, Italy	risk of breast cancer (–); concentration of circulating levels of C-reactive protein (↑)
Koelman et al. [51]	6.8 years	N = 636; age 50.8 ± 8.1 years *, Germany	the concentrations of inflammatory biomarkers (–)

N—number of participants; *—age at baseline; (↓)—decreased; (–)—no statistically significant association; (↑)—increased; NHS—Nurses’ Health Study; HPFS—Health Professionals Follow-Up Study; CVD—cardiovascular diseases; WC—waist circumference; BMI—body mass index; CHD—coronary heart disease.

**Table 2:** Main results from cross-sectional studies concerning the association between the Planetary Health Diet with non-communicable diseases, risk factors, and cause-specific and total mortality.

References	Characteristics of the Study Group	Results: The Planetary Health Diet vs.:
de Oliveira Neta et al. [52]	N = 398; adults and elderly, Brazil	total cholesterol level, LDL-C, SBP, risk of self-reported diabetes, risk of dyslipidemia (↓) HDL-C, TG, FBG, risk of self-reported hypertension (–)
Cacau et al. [53]	N = 14,155, age 35–74 years, Brazil	total cholesterol level, LDL-C, non-HDL-C, SBP, DBP (↓); HDL-C, TG, HOMA-IR (–)
Frank et al. [54]	N = 8128 for the laboratory-based sample (age 48.6 ± 15.6 years) and N = 3933 for the fasted subsample, USA	fasting TG, WC, predicted probability of high WC, predicted probability of high blood pressure, predicted probability of low HDL-C, (↓); HDL-C (↑) SBP, DBP, FPG, predicted probability of high FPG, predicted probability of high fasting TG (–)
McDowell et al. [55]	N = 3458; median age 64 years (Q1–Q3: 60–69 years), Australia	lower values of the EAT-Lancet Index in the group with high blood pressure (ever and current) vs. no hypertension lower values of the EAT-Lancet Index in the group with diabetes (ever) vs. no diabetes no significant results in the groups with cancer, high cholesterol, diabetes, kidney disease, COPD, CVD vs. the groups without those abnormalities
Cacau et al. [56]	N = 14,515, adults (34–59 years) and elderly (≥60 years), Brazil	BMI and WC values, risk of overweight, obesity, having increased WC or substantially increased WC (↓)
Macit-Çelebi et al. [57]	N = 1112; mean age 28.7 years (SE = 0.34), Turkey	BMI values (↓)
Ambroży et al. [58]	N = 216, median age 30.0 years (Q1–Q3: 23.0–40.0 years), Poland	lower percentage of overweight/obesity participants in the group who followed the PHD vs. the group who did not follow the PHD
Shojaei et al. [59]	N = 5206; age 45.57 ± 9.01 years, Iran	prevalence of metabolic syndrome, hypo-HDL cholesterolemia, abdominal obesity (↓)

N—number of participants; (↓)—decreased; (–)—no statistically significant association; (↑)—increased; LDL-C—LDL-cholesterol level; SBP—systolic blood pressure; HDL-C—HDL-cholesterol level; TG—triglycerides level; FBG—fasting blood glucose; DBP—diastolic blood pressure; HOMA-IR—Homeostatic Model Assessment for Insulin Resistance; WC—waist circumference; FPG—fasting plasma glucose; BMI—body mass index; COPD—chronic obstructive pulmonary disease; CVD—cardiovascular disease; PHD—Planetary Health Diet.

## Data Availability

No new data were created or analyzed in this study. Data sharing is not applicable to this article.

## Supplementary Materials

The following supporting information can be downloaded at: https://www.mdpi.com/article/10.3390/foods14223909/s1, Figure S1: Flowchart with the strategy of literature search.

## Abbreviations

The following abbreviations are used in this manuscript:

BMI	body mass index
CHD	coronary heart disease
COPD	chronic obstructive pulmonary disease
CVD	cardiovascular diseases
DBP	diastolic blood pressure
EPIC	European Prospective Investigation into Cancer and Nutrition
FBG	fasting blood glucose
FPG	fasting plasma glucose
GHG	Greenhouse gas
HDL-C	high-density lipoprotein cholesterol
HOMA-IR	Homeostatic Model Assessment for Insulin Resistance
HPFS	Health Professionals Follow-Up Study
IARC	International Agency for Research on Cancer
LDL-C	low-density lipoprotein cholesterol
MD	Mediterranean Diet
NHS	Nurses’ Health Study
PHD	Planetary Health Diet
SBP	systolic blood pressure
TG	triglycerides
UK	United Kingdom
WC	waist circumference
