# Supplementary figures and images for: The Role of a Sustainable Planetary Health Diet in the Prevention of Non-Communicable Diseases and Cause-Specific Mortality: A Narrative Review
Source: Foods. 2025 Nov 15;14(22):3909. doi: 10.3390/foods14223909 (PMC12651933; doi:10.3390/foods14223909)

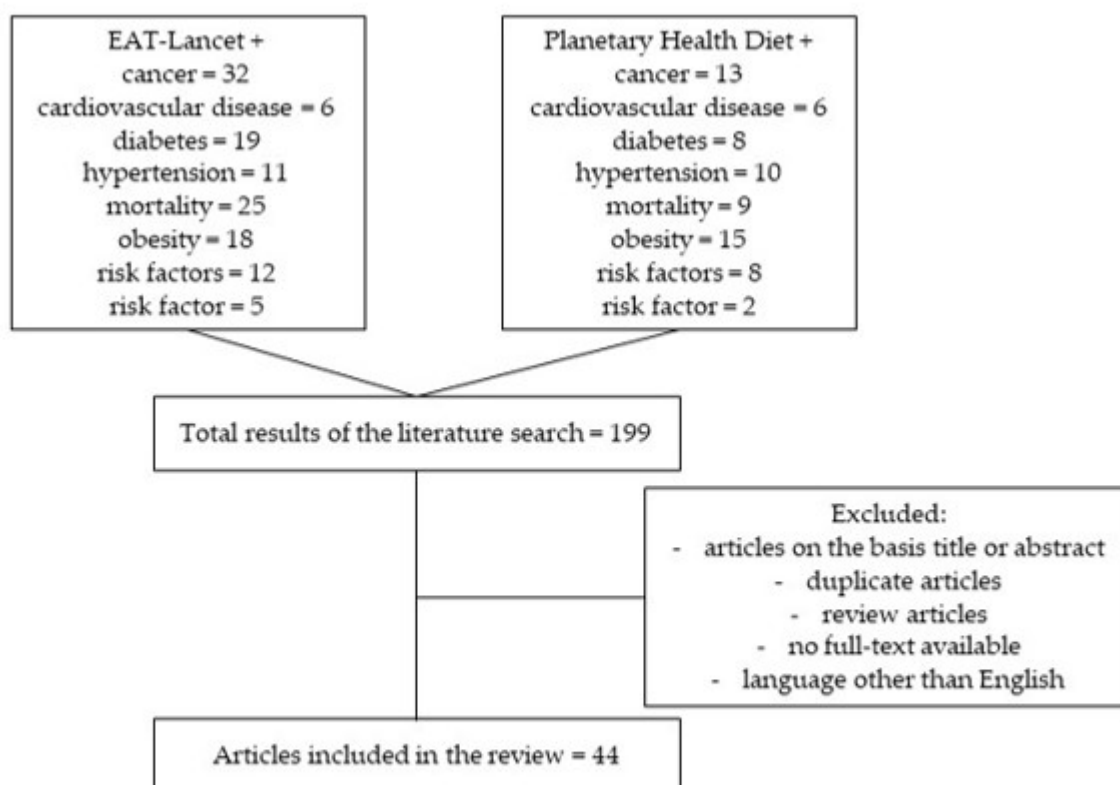

Figure S1. Flowchart with the strategy of literature search

Supplement: Supplementary file 1 [file foods-14-03909-s001.zip › foods-3930246-supplementary.pdf]
